# Popular Science Monthly for October

**Published:** 1877-10-20

**Authors:** 


Popular Science Monthly for October.—By E. L. & W. J. Youman's, comes filled with its usual amount of interesting and instructive reading matter. D. Appleton & Co., 549 Broadway, N. Y.

